# Precision shaping of elastic stable intramedullary nail for the treatment of metaphyseal diaphysis junction fracture of the distal radius in children: a preliminary report in two centers

**DOI:** 10.1186/s12891-023-06332-x

**Published:** 2023-03-29

**Authors:** Liu Chaoyu, Jia Guoqiang, Xu Wenqiang, Meng Lian, Shan Jing, Liu Yong

**Affiliations:** 1 The People’s Hospital of Fuyang City, Fuyang, 236011 China; 2 Department of Orthopedics, Children’s Hospital of Fudan University Anhui Hospital, Hefei, 230051 China

**Keywords:** Metaphyseal diaphysis junction, Radius, Elastic stable intramedullary nail, Retrograde, Children

## Abstract

**Background:**

This study introduces a novel retrograde precision shaping elastic stable intramedullary nailing (ESIN-RPS) technique and reports clinical outcomes in pediatric distal radius metaphyseal diaphysis junction (DRMDJ) fracture.

**Methods:**

Data about DRMDJs were collected from February 1, 2020, to April 31, 2022 at two hospitals, retrospectively. All patients were treated with closed reduction and ESIN-RPS fixation. The operation time, blood loss, fluoroscopy times, alignment, and residual angulation on X-ray were recorded. At the last follow-up, the function of wrist and forearm rotation were evaluated.

**Results:**

Totally, 23 patients were recruited. The mean time of follow-up was 11 months and the minimum was 6 months. The mean operation time was 52 min, and the mean fluoroscopies pulses were 6 times. The postoperative anterioposterior (AP) alignment was 93 ± 4% and the lateral alignment was 95 ± 3%. The postoperative AP angulation was (4 ± 1)°, and the lateral angulation was (3 ± 1)°. At the last follow-up, the evaluation of the Gartland and Werley demerit criteria of wrist revealed 22 excellent cases and 1 good case. The forearm rotation and thumb dorsiflexion functions were not limited.

**Conclusion:**

The ESIN-RPS is a novel, safe, and effective method for the treatment of pediatric DRMDJ fracture.

## Introduction

The radius is one of the most common fracture sites of upper extremities in children and represents 80% of forearm fractures [1]. The distal radius metaphyseal diaphysis junction (DRMDJ) fracture, first proposed by Lieber in 2010, is a special type of fracture and differs from metaphyseal fracture and radial shaft fracture [2–4]. DRMDJ is characterized by a specific anatomy: (1) the site includes part of the tendon muscle migration and lacks significant muscle attachment on the bone surface; (2) there are less vascular perforations than the metaphyseal or shaft part; and (3) the proximal medullary cavity gradually expands toward the distal end [3, 4]. For stable DRMDJ fractures, conservative treatment is recommended [5]. However, in some cases, due to unstable fractures, poor alignment after closed reduction, and secondary displacement, the surgical treatment is required [6–8]. In addition, due to the anatomical characteristics of the diameter of the radial cavity, DRMDJ fractures are easily displaced laterally because of unsuitable fixation [9]. Furthermore, the remodeling ability is limited in lateral displacement due to inconsistent direction of the joint activity according to the Blount principle in DRMDJ fracture, and this easily causes to complications [10–13].

The traditional surgical treatments for DRMDJ fracture include the closed reduction with Kirschner wire fixation, the elastic stable intramedullary nailing (ESIN) fixation, the open reduction with plate fixation, and the external frame fixation. But all the methods above have disadvantages [6–8, 11–14]. Thus, in this study we propose a new method of retrograde precision shaping ESIN (ESIN-RPS) to treat the DRMDJ fracture and report the preliminary outcomes.

## Materials and methods

### Patients

This study was approved by the institutional review board of the Children’s Hospital of Fudan University Anhui Hospital (EYLL-2019-035) or People’s Hospital of Fuyang City (FYRMH-LL-20,200,190); and was performed in accordance with the Declaration of Helsinki. Consent was obtained from the patients and their guardians.

The inclusion criteria were (1) age ≤ 14 years; (2) DRMDJ fracture with closed reduction failure; (3) follow-up time of more than 6 months. The exclusion criteria were (1) combining with obvious ulnar fracture displacement requiring surgical treatment; (2) multiple fractures of the ipsilateral limbs; (3) open fractures;4) and iterative fractures.

**Fig. 1:**
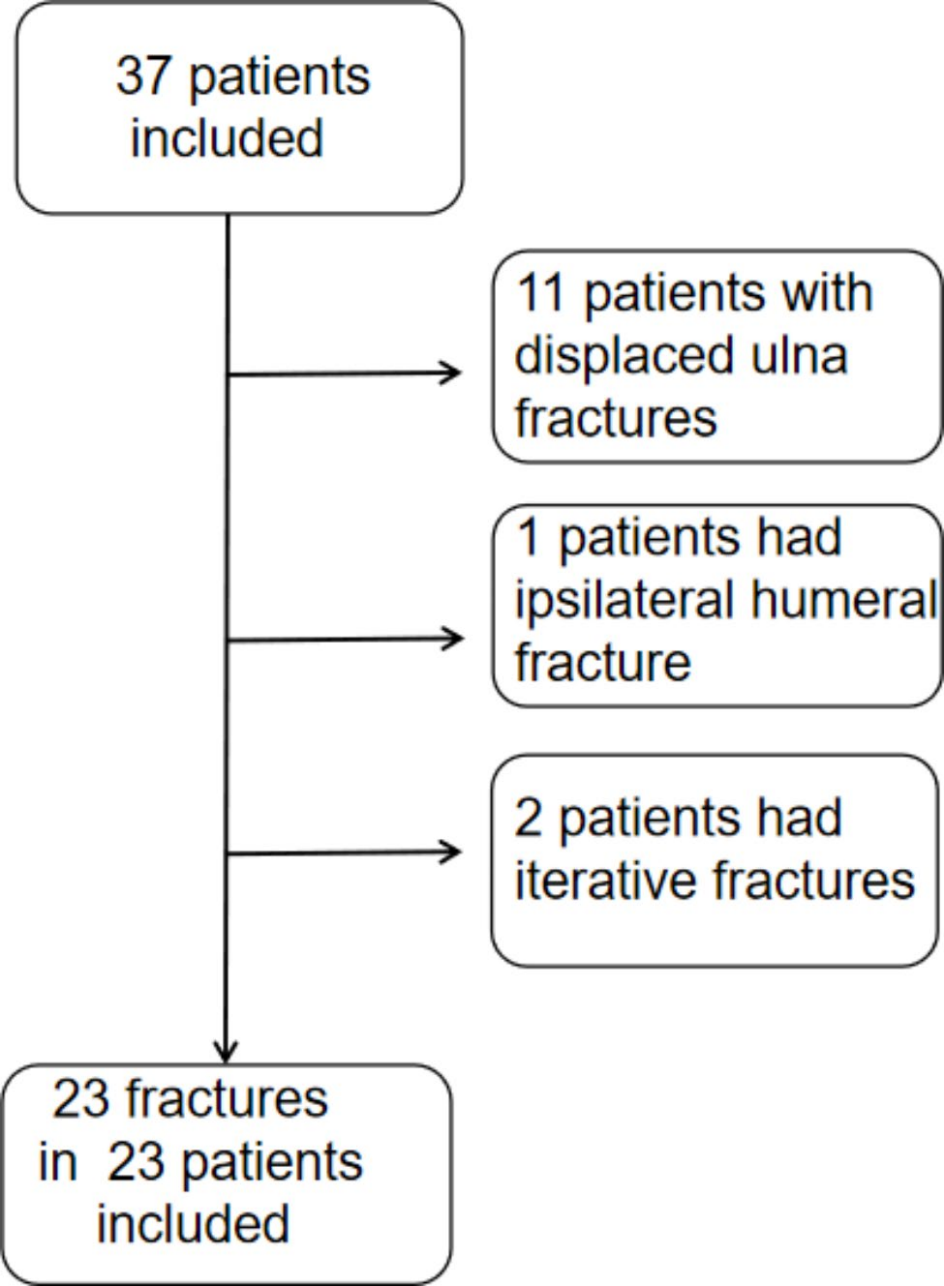
Flow chart of patient selection. Initially, 37 fractures were found, 14 patients were excluded, and the final patient cohort was composed of 23 fractures after application of exclusion criteria

In total, 23 patients from February 2020 to April 2022 were enrolled (Fig. 1), including 17 males and 6 females; the mean age was 8 ± 3 years (range: 5–14 y). There were 12 cases on the left side and 11 cases on the right side. Two cases consisted of a pure radial fracture and 21 were combined with greenstick ulnar fracture; the mean time from injury to operation was 4 days (range: 1–15 d).

### Preoperative design

**Fig. 2:**
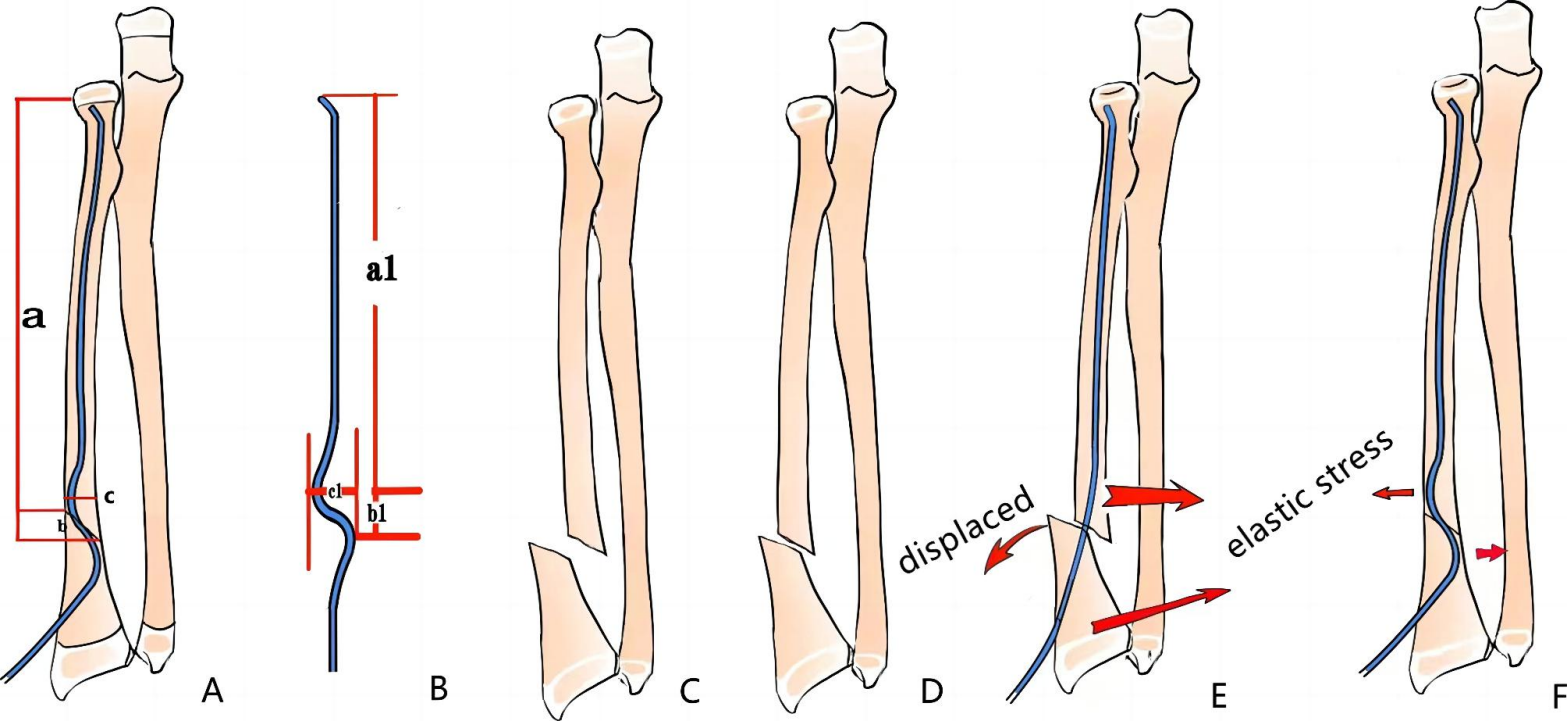
A: the distance between the proximal fracture site and the physeal plate of the proximal radius was accurately measured preoperatively and was denoted as “a”; the length of the fracture line relative to the longitudinal axis of the radial shaft was denoted as “b”; the diameter of the medullary cavity at the fracture site was “c.” B: the distance between the head of ESIN and the apex of the proximal prebending point was “a_1_”; the longitudinal distance between the two apexes of the prebending point was “b_1_”; and the transverse distance was “c_1_”. C: DRMDJ fracture with obvious displacement. D: The degree of displacement decreases after closed reduction. E: Inserting ESIN with conventional method, the degree of displacement increased. F: Inserting the precision shaping ESIN, the satisfied fracture alignment is obtained

The sizes of intramedullary titanium nails were selected according to the two-third diameter of the narrowest part of medullary cavity on the X-ray. As shown in Fig. 2, the distance between the proximal fracture site and the physeal plate of the proximal radius was accurately measured using a picture archiving and communication system preoperatively and was denoted as “a”; the length of the fracture line relative to the longitudinal axis of the radial shaft was denoted as “b”; the diameter of the medullary cavity at the fracture site was “c”; the distance between the head of ESIN and the apex of the proximal prebending point was “a_1_”; the longitudinal distance between the two apexes of the prebending point was “b_1_”; and the transverse distance was “c_1_”. To avoid damaging the physis and achieve a satisfactory reduction, the following conditions were met: a_1_ was 2 mm less than a, b_1_ was 2 mm greater than b, and c_1_ was equal to or slightly greater than c. According to the above conditions, the precision measurement and shaping of the ESIN was performed using a sterile scale before insertion. Note that the two apexes of the prebending point were placed in the same plane to avoid lateral displacement of the fracture when the nail was inserted.

### Surgical techniques

**Fig. 3:**
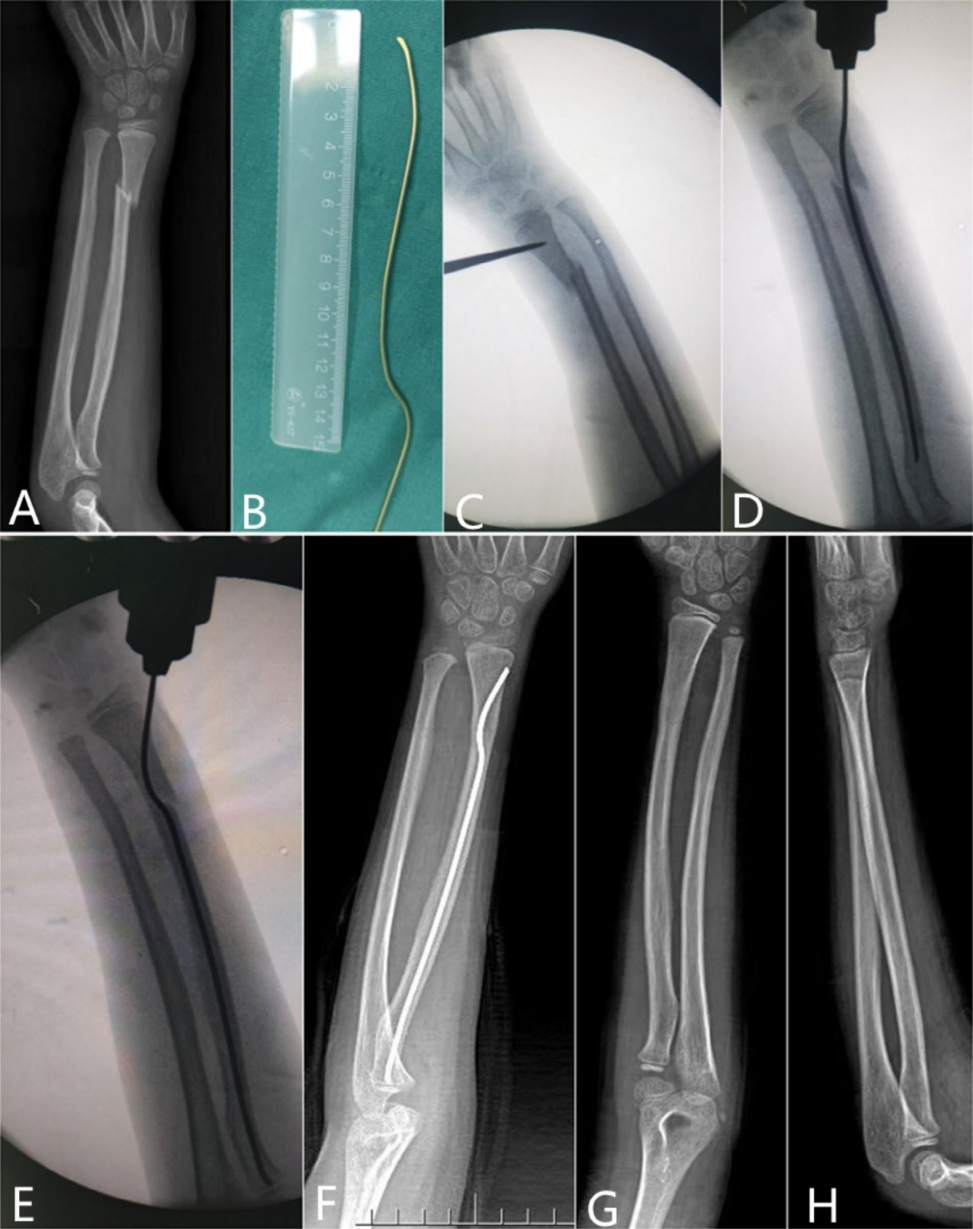
A: seven-year-old girl presented with a left DRMDJ fracture. B: According to the preoperative X-ray measurement, the precision shaping of ESIN based on fracture characteristics was performed. C: Intraoperative localization of the insertion point. D: The displacement of distal fragment was aggravated when a non-precision shaping ESIN was inserted directly. E: Insertion of a precision shaping ESIN for accurate reduction. F: The fracture healed well three months after the operation. G&H: The radius healed completely six months after the operation on AP as well as lateral films, and the ESIN was removed

The operation was performed by two attending surgeons. After general anesthesia, the important glands were protected by regular lead clothing. The length of incision was about 1.5 cm in the distal radius. Via fluoroscopy, the entry point was determined around the classical Lister node; then, the precision shape ESIN was inserted through the point and placed proximally to the physeal plate by 0.5 to 1 cm. Closed reduction of the fracture allowed to obtain better alignment. The surgeon maintained the fracture side with one hand and held the ESIN handle with the other hand, then slowly inserted the nail until the proximal prebending vertex on the diaphyseal fracture side. The ESIN passed through the fracture site and the distal vertex of the nail prebending was located at the distal end of the fracture line. Next, the quality of reduction could be assessed on fluoroscopy, and the stability of the distal fragment could be checked. The tail of ESIN was then cut off and located on the surface of the deep fascia to prevent tendon injury. The procedures were shown in Fig. 3. A short arm plaster was used at the 90° flexion of the elbow joint.

The anteroposterior (AP) and lateral fracture alignment = (actual contact surface length of two fragments / diameter of fracture fragment) * 100% on X-ray films. The plaster was removed after four weeks and the ESIN was removed after 6 months. At the last follow-up, the secondary displacement, refracture, nonunion, limited forearm rotation, and wrist function according to Gartland and Werley demerit criteria were assessed [14].

### Statistical analysis

Statistical analysis was performed using SPSS version 24.0 (IBM, Armonk, New York, United States). Statistical methods included standard descriptive summaries of demographic data and were expressed as mean and standard deviation.

## Results

### Perioperative data

The perioperative data were shown in Table 1. The mean operation time was 52 ± 11 min (range: 38–71 min), the mean intraoperative bleeding volume was 6 ± 3 mL (range: 3–11 ml), the mean number of fluoroscopies was 6 ± 3 times (range: 3–11 times), and the mean length of incision was 1 ± 1 cm (range: 1.2–1.9 cm). All children had anatomical or nearly anatomical reduction. The mean postoperative AP alignment rate was 93 ± 4%, and the mean lateral alignment rate was 95 ± 3%. The mean angulation on AP plane was 4 ± 1°, and the mean angulation on lateral plane was 3 ± 1°.

**Table 1:** Perioperative data

Parameters	Results (Mean ± SD)
Age (year)	8 ± 3
Duration of operative time (min)	52 ± 11
Incision length (cm)	1 ± 1
Blood loss (mL)	7 ± 3
Fluoroscopy (times)	6 ± 3
Hospitalization costs (RMB, thousand)	14 ± 1
Hospitalization days (d)	4 ± 3
Pre-operation	
Alignment on AP plane (°)	35 ± 4
Alignment on lateral plane (°)	26 ± 9
Angulation on AP plane (°)	23 ± 6
Angulation on lateral plane (°)	42 ± 15
Shortening (mm)	12 ± 4
Post-operation	
Alignment on AP plane (°)	93 ± 4
Alignment on lateral plane (°)	95 ± 3
Angulation on AP plane (°)	4 ± 1
Angulation on lateral plane (°)	3 ± 1

SD: Standard Deviation

### Outcomes and complications

The mean time of follow-up was 11 ± 3 months (range: 6–16 m). At the latest follow-up, there was no secondary displacement, delayed union or nonunion of the fracture on X-ray. There was no limited forearm rotation, the Gartland and Werley demerit criteria were excellent in 22 cases and good in 1 case. Four patients had skin irritation, and no patient had superficial or deep infection, refracture, or rupture of the extensor pollicis longus tendon.

## Discussion

DRMDJ fractures are located at a tendon–muscle junction part of the radius. The healing ability of the fracture is relatively poor and may cause nonunion problems [3, 12, 13]. This study pioneered the use of retrograde ESIN for precise shaping fixation and accurate measurement of the DRMDJ fracture before surgery and provided a reasonable choice for surgeons. Compared with a previous purely de- elastic retrograde fixation study from the same incision, our intervention greatly improved the stability of radius reduction, added the alignment rate of fracture, reduced the risk of secondary displacement of the distal fragment, and successfully addressed the early rehabilitation of patients [6].

There are some innovations characterizing this novel ESIN-RPS surgical technique: (1) the method allows strict planning according to the preoperative X-ray films of the patients, precisely shaping of the ESIN and directly fixation of the fracture after reduction to shorten the operation time and reducing intraoperative blood loss, infection, and radiation exposure; (2) the ESIN nail is accurately bent at the fracture site to avoid lateral displacement of the distal fracture fragment when inserting the nail. The ESIN completely enters the radius and the two prebending apexes hug the radial medullary cavity. With the ESIN touching at the distal and proximal ends of fracture line, a stable “four-point support” is achieved. The four-point are: the first one is close to but not touching the physeal plate of radius; the second and third ones are the pre-bended apexes; the fourth one is the insertion point; (3) good alignment of fracture is conducive to fracture healing and early functional exercise; (4) the entry point avoids the physeal plate, thus decreases the possibility of physeal injury; (5) the tail of ESIN is located at the surface of the deep fascia and dose not contact with the extensor pollicis longus tendon directly, which reduces the possibility of tendon abrasion.

Compared with other techniques, this novel ESIN-RPS with minimally invasive technique allowed to return to the exercise for the early rehabilitation function, return to school life earlier, address fear issues, and reduce family and social costs [6–8, 15]. For DRMDJ fractures, it is very difficult to directly fix the fracture with crossed K-wires because of limited angulation of insertion [2, 8, 13]. The further the fracture line from the distal physeal plate of radius, the more difficult to fix a well-placed K-wires [8]. Therefore, some surgeons insert K-wires through the physeal plate for intramedullary fixation. It is difficult to place the K-wires which requires multiple insertions and fluoroscopies, which increases radiation exposure and physeal plate injury and could leads to iatrogenic premature closure of the distal radius physis [15]. Then, for reducing the iatrogenic premature closure risk, a mini-invasive incision in the physis and K-wire fixation has also been proposed with limited insertion times [16]. However, a pin still is inserted across the physis to cause damage on the physeal plate. Li et al. compared the treatment outcomes of external frame and K-wires fixation, indicating that the external frame achieved a shorter operative time, less tendon irritation, and better radiographic outcomes [8]. However, the cost, cosmetics, infection and the risk of delayed union cause dissatisfaction of the parents. ESIN could reduce these risks in the daily practice. The anterograde ESIN has been used in a complicated approach and requires hardware removal with deeper muscle dissection, which leads to a higher risk of posterior interosseous nerve injury [7]. Meanwhile, soft tissue damage is significant, and the tip of the nail continues to wear the supinator muscle, affecting forearm rotation [7]. Cai et al. attempted retrograde ESIN in traditional entry technique, the proximal fragment was often pushed to the opposite side by the nail because of the short distal fracture fragment, and it could result in unsatisfied alignment and unstable fixation of the fracture [6]. In this new ESIN-RPS technique, the “four-point support” is achieved and avoids the lateral displacement problem with stable fixation. Krohn et al. introduced a similar surgical technique note but without functional follow-up [17]. This study added a short-term follow-up outcomes of the new surgical technique, proving that it was an effective procedure.

It was slightly difficult when removing the hardware because of the increased friction between the ESIN and the medullary cavity. In this study, we found leaving the outside tail of the nail long enough can reduce the difficulty of removing out.

Age is a significant factor in treating DRMDJ. For children under 7 years old, conservative treatment can often obtain good results in stable fracture. However, the body size of patients, fracture characteristics, family requirements etc. might also affect treatment method choice. There were three 6-year-old patients and one 5-year-old in this study. In these four patients, the fracture lines were short oblique fractures, and the displacement of the fractures were obviously unstable in initial radiography. Closed reduction were firstly chose and failed. Due to the requirement of their patients, we performed the operation.

There are some limitations in this study which should be considered. Firstly, the study involved a small number of patients and was a retrospective design. Secondly, this study did not have a longer follow-up to review the problems about this noval technique. Thirdly, this intervention requires an accurate measurement and shape of ESIN before operation, which may have a long learning period.

In conclusion, the ESIN-RPS technique is a minimally invasive, effective, and safe method with fewer postoperative complications for the treatment of pediatric DRMDJ fractures.

## Data Availability

The datasets generated and/or analysed during the current study are not publicly available due further research is not completed but are available from the corresponding author on reasonable request.

## Declarations

### Statement of study participation

Consent was obtained from the patients or their guardians in Chinese.
